# Relationship between Inflammatory Cytokine and Depressive Symptoms in Postpartum Women: A Systematic Review

**DOI:** 10.34763/jmotherandchild.20252901.d-25-00006

**Published:** 2025-07-19

**Authors:** Ardesy Melizah Kurniati, Radiyati Umi Partan, Peby Maulina Lestari, Iche Andriyani Liberty, Mohammad Zulkarnain, Kemas Yusuf Effendy, Bima Indra

**Affiliations:** Department of Nutrition, Faculty of Medicine, Universitas Sriwijaya, Palembang, South Sumatera, Indonesia; Division of Rheumatology, Department of Internal Medicine, Dr. Mohammad Hoesin General Hospital, Faculty of Medicine, Universitas Sriwijaya, Palembang, South Sumatera, Indonesia; Division of Maternal-Fetal Medicine, Department of Obstetrics and Gynaecology, Dr. Mohammad Hoesin General Hospital, Faculty of Medicine, Universitas Sriwijaya, Palembang, South Sumatera, Indonesia; Department of Public Health and Community Medicine, Faculty of Medicine, Universitas Sriwijaya, Palembang, South Sumatera, Indonesia; Faculty of Medicine, Universitas Sriwijaya, Palembang, South Sumatera, Indonesia

**Keywords:** postpartum depression, inflammation, cytokine

## Abstract

**Introduction:**

The appearance of depressive symptoms is a prevalent mental health issue among women, with inflammatory cytokines being explored as potential biomarkers. This systematic review evaluates the relationship between inflammatory cytokines and depressive symptoms in postpartum women.

**Material and methods:**

Following PRISMA 2020 guidelines, a search was conducted across five electronic databases (PubMed, ScienceDirect, CINAHL, Web of Science, and Tripdatabase) up to September 2024. Studies examining the relationship between inflammatory cytokines and postpartum depressive symptoms that were published in English were included. The risk of bias was assessed using the Revised Risk of Bias Assessment Tool for Non-Randomised Studies of Interventions 2.

**Results:**

Nine studies were included in this review. A total of 888 postpartum women were analysed across nine studies. IL-1β was significantly elevated in postpartum women with depressive symptoms in two studies. IL-6 showed mixed findings, with three studies supporting an association, while three others did not. IL-10 and TNF-α generally showed no significant relationship with depressive symptoms. The overall quality of the studies included varied, with three studies at high risk of bias and five at low risk.

**Conclusion:**

Evidence on the relationship between inflammatory cytokines and postpartum depressive symptoms is inconsistent. IL-1β may be linked to depressive symptoms. TNF-α and CRP may have no relationship with depressive symptoms in postpartum women, but the roles of IL-6 and IL-10 remain unclear. More high-quality research is necessary to determine the clinical significance of cytokine levels in predicting or managing postpartum depression.

## Introduction

Postpartum depression (PPD) is a significant mental health issue that affects a substantial number of new mothers globally. The prevalence of PPD varies widely, with estimates ranging from 10% to 70%, depending on socioeconomic factors, cultural contexts, and social support. In low- and middle-income countries, the prevalence can reach 19.2%, while it is around 12.9% in high-income countries. [1] This disparity highlights the influence of socio-environmental factors on PPD incidence. PPD negatively affects not only the mothers but also child development and family dynamics. Children of mothers with PPD are at risk for behavioural issues and cognitive delays. [2] Additionally, PPD may impair breastfeeding practices, leading to early cessation and negatively impacting infant health. [3,4] PPD's psychosocial consequences include maternal isolation, feelings of inadequacy, and the intensification of depressive symptoms. Social support plays a crucial role in reducing the incidence and severity of PPD. [5] Intervention strategies focused on counselling and social support programs have shown promise in mitigating PPD. [6] Furthermore, stressful life events and conditions such as preterm birth can increase PPD risk, with some studies indicating a prevalence of 70% among mothers of preterm infants. [7] PPD is often a dyadic phenomenon affecting both parents, and family-centred interventions are essential for addressing its broader impact. [8] Psychotherapy, including cognitive-behavioural therapy (CBT), and pharmacotherapy, such as SSRIs, have been found effective in treating PPD. [9] Increasing awareness and training healthcare providers to identify PPD can lead to improved outcomes. [10]

Depressive symptoms in postpartum women arise from a multifactorial aetiology involving hormonal, neurobiological, and psychosocial factors. The sharp decline in estrogen and progesterone following childbirth is associated with the onset of depressive symptoms in new mothers. [11] Dysregulation of the hypothalamic-pituitary-adrenal (HPA) axis and elevated cortisol levels have also been linked to stress and depressive symptoms in postpartum women. [12] Additionally, neuroactive steroids, such as allopregnanolone, and neuroinflammatory processes may disrupt neurotransmitter systems like serotonin and dopamine, contributing to mood disturbances. [13] Psychosocial stressors, such as life events or lack of social support, also increase vulnerability to PPD, underscoring the importance of a biopsychosocial model in understanding it. [14]

Inflammatory cytokines, such as IL-6, TNF-α, and CRP, play a key role in depressive symptoms by affecting neurotransmitter metabolism and neurogenesis. [15,16] Elevated pro-inflammatory cytokine levels have been observed in individuals with depression, which suggests a bidirectional relationship where inflammation contributes to depression, and depression, in turn, exacerbates inflammatory responses. [17,18] These cytokines may also reduce reward system responsiveness in the brain, leading to anhedonia, a core symptom of depression. [19,20] Anti-inflammatory treatments are being explored as adjunct therapies for depression, especially in individuals with elevated inflammation. [21,22]

Research has demonstrated a link between pro-inflammatory cytokines and PPD. Elevated levels of IL-6 and TNF-α during the postpartum period are correlated with increased depressive symptoms. [23,24] These cytokines may influence neurotransmitter regulation and neuroplasticity, leading to mood disturbances. Additionally, psychosocial stressors, such as physical trauma during childbirth, can trigger inflammatory responses, contributing to PPD. [25] Genetic factors may further modulate the inflammatory response, increasing the susceptibility to PPD. [25]

Despite increasing evidence linking inflammatory cytokines to PPD, the research presents several inconsistencies and gaps. Studies on the role of specific cytokines, such as IL-6 and TNF-α, show conflicting results, with some finding significant associations while others report no relationship. Furthermore, many studies lack large and diverse sample sizes, which limits the generalizability of their findings. Another challenge is the variation in cytokine measurement techniques and the timing of assessments across studies, making it difficult to draw definitive conclusions. Additionally, few studies consider the interplay between biological and psychosocial factors, such as stress and social support, that may influence both cytokine levels and depressive symptoms. This fragmented evidence underscores the need for a comprehensive synthesis of the available data to clarify the role of inflammatory cytokines in PPD and identify potential biomarkers for early detection and intervention.

The objective of this systematic review is to evaluate the relationship between inflammatory cytokines and depressive symptoms in postpartum women. By synthesising data from existing studies, this review aims to provide a clearer understanding of the role of cytokines in the pathophysiology of PPD. Specifically, this review aims to identify which cytokines, if any, are consistently associated with postpartum depressive symptoms and to highlight areas where further research is needed. This analysis will help inform future studies and potentially contribute to the development of targeted interventions for PPD.

## Material and methods

This systematic review was conducted in accordance with the PRISMA 2020 guidelines. [26] A comprehensive literature search was performed in five electronic databases: PubMed, ScienceDirect, CINAHL, Web of Science, and Tripdatabase. The search included studies published in English up to September 2024. Keywords and medical subject headings (MeSH) related to “inflammatory cytokines”, “postpartum blues”, and “postpartum depressive symptoms” were used. The reference lists of included studies were also manually screened to identify additional relevant studies.

Studies were included if they examined the relationship between inflammatory cytokines and depressive symptoms in postpartum women. Eligible studies had to involve human participants, focus on postpartum women, and include assessments of both inflammatory cytokine levels and depressive symptoms. Exclusion criteria were non-English language articles, studies that did not measure both variables of interest, and studies involving non-postpartum populations.

Two reviewers independently extracted data, including study characteristics, participant demographics, types of cytokines measured, and assessments of depressive symptoms. Disagreements were resolved by discussion until a consensus was reached. The primary outcome was the association between cytokine levels and depressive symptoms.

The risk of bias in the included studies was evaluated using the Revised Risk of Bias Assessment Tool for Nonrandomized Studies of Interventions (RoBANS 2). [27] Domains assessed included comparability of target groups, selection, confounders, blinding of assessors, and outcome reporting.

## Results

**Figure 1.**
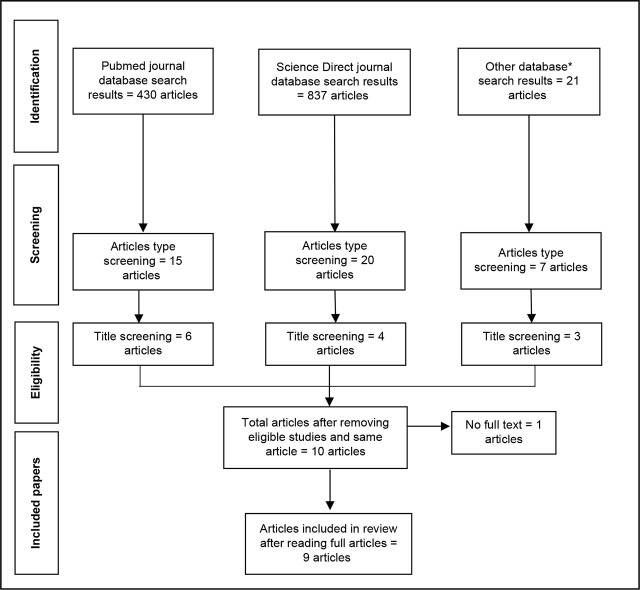
PRISMA flowchart.

The initial search identified a total of 1,308 articles across five electronic databases. After filtering based on original research article type, 42 articles were screened based on titles and abstracts. Of these, 13 articles were selected for full-text review. Following a full-text assessment, two studies were removed as duplicates, and one study was excluded because it lacked full-text access. [28] The nine (9) studies met the eligibility criteria and were included in this systematic review. The PRISMA flowchart illustrating this search process is shown in Figure 1.

The risk of bias assessment revealed variability in study quality. Using the Revised Risk of Bias Assessment Tool for Nonrandomized Studies of Interventions (RoBANS 2), three studies were classified as having a high risk of bias, primarily due to issues with selection bias and selective outcome reporting. Conversely, five studies were rated as having a low risk of bias, demonstrating robust methodologies and thorough reporting. Detailed risk of bias results are summarised in Table 1.

**Table 1.** Risk of Bias Assessment using Revised Risk of Bias Assessment Tool for Nonrandomised Studies of Interventions (RoBANS 2).

**No.**	**Domain**	**Study Index Number**								
		**1**	**2**	**3**	**4**	**5**	**6**	**7**	**8**	**9**
1.	Comparability of the target group	Low	High	Unclear	Unclear	Low	Low	Low	Low	Low
2.	Target group selection	Low	Low	Low	Low	Low	Low	Low	Low	Low
3.	Confounders	Low	High	Low	Low	Low	Low	Unclear	Low	Low
4.	Measurement of exposure	Low	Low	Low	Low	Low	Low	Low	Low	Low
5.	Blinding of assessors	Low	Low	Low	Low	Low	Low	Low	Low	Low
6.	Outcome assessment	Low	Low	Low	Low	Low	Low	Low	Low	Low
7.	Incomplete outcome data	Low	Low	Low	Low	Low	Low	Low	Unclear	Low
8.	Selective outcome reporting	High	Low	High	High	Low	Low	Low	Low	Low

**Overall results**		**Moderate**	**High**	**High**	**High**	**Low**	**Low**	**Low**	**Low**	**Low**

The nine studies included a total of 888 postpartum women. Study designs varied, with seven cohort studies, one case-control study, and one cross-sectional study. The majority of studies (four) were conducted in the United States, while one study each came from Canada, Poland, Sweden, China, and Taiwan. The average age of participants ranged from 25 to 31 years. Depressive symptoms were assessed using the Edinburgh Postnatal Depression Scale (EPDS) in five studies, the Centre for Epidemiologic Studies Depression Scale (CES-D) in two studies, and the Hamilton Depression Rating Scale (HDRS) in two studies.

Analysis of the data revealed that IL-1β was significantly elevated in postpartum women with depressive symptoms, supported by two studies, with one study having a low risk of bias and the other one having a high risk of bias. Inconsistent findings were observed for IL-6, with three studies supporting an association and three opposing it. IL-10 and TNF-α generally showed no significant association with depressive symptoms. Specifically, IL-10 had one supportive study and two opposing studies, whereas TNF-α was not significantly associated in any of the included studies. CRP levels showed no significant relationship with depressive symptoms, with one study supporting and three studies refuting the association. A summary of the included studies is presented in Table 2.

**Table 2.** Summary of included studies.

**No.**	**Study details**	**Subject characteristics**	**Result**
1.	Cheng, CY et al.^31^ Taiwan, 2014 Cohort	12 pregnant women ≥ 17 years, without pregnancy complications, over 36 weeks of gestation, and who understood English (34 participants were not complete in the study). Maternal stress was assessed using PSS, and Maternal depressive symptoms were assessed using CES-D.	Levels of prenatal cytokines (IL-1, IL-5, IL-7, MPI-1α, GM-CSF, MCP, MIB) were not statistically different when participants were grouped as either low-high stress, yes-no fatigue, or yes-no depression.
2.	Brann, Emma, et al.^32^ Sweden, 2018 Case-control	A total of 169 women ≥18 years of age with gestational age 17 to 32 weeks, do not have confidential personal information, speak Swedish, and are scheduled for a routine pregnancy ultrasound at Uppsala University Hospital. Depressive symptoms were assessed using EPDS. Mean age was 30.5 + 4.9 years	Five cytokines were significantly elevated in women with postpartum depressive symptoms; TRANCE, HGF, IL-18, FGF-23, and CXCL1.
3.	Groer, Maureen et al.^33^ United States, 2014 Cohort	72 healthy pregnant women with gestational age 15 to 25 weeks were followed for six postpartum months with immune and hormone measures, dysphoric moods, and stress scales. Mood and stress were measured with POMS and PSS.	No significant relationships were found between inflammatory cytokines (hs-CRP, IFN-γ, TNF-α, IL-2, IL-6) and psychosocial measures.
4.	Corwin, Elizabeth, et al.^34^ United States, 2014 Cohort	38 healthy postpartum women were recruited within 24 hr (Day 0) of giving birth to a singleton infant via vaginal delivery. Inclusion criteria were that births were without complications, including maternal haemorrhage, transfusion, or surgery, and that both mothers and infants left the hospital within 72 hr. Maternal depressive symptoms were assessed using the CES-D on day 28 postpartum. Mean age was 27.7 + 4.8 years	Women with depressive symptoms on Day 28 had elevated levels of IL-1β 2 weeks previously (on Day 14) compared to women without symptoms of depression on Day 28. There were no differences in IL-6 levels at any time based on the level of depressive symptoms on Day 28.
5.	Sha, Qiong, et al.^35^ United States, 2022 Cohort	114 pregnant women were enrolled in their first trimester, returning each trimester and in the postpartum period for psychiatric assessments and blood sampling. The inclusion criteria for enrollment were pregnant women aged 18 years or older. Exclusion criteria were: nonpregnant women and patients with psychotic symptoms and/or severe cognitive impairment. Depressive symptoms were assessed using EPDS. Mean age was 25 + 5.9 years	IL-1β, IL-6, and QUIN were significantly associated with depression severity and/or higher odds of having an EPDS ≥ 13. IL-2, IL-8, IL-10, TNF, TRY, SERO, KYN, KYNA, PICO, rKT, rQK, and rQP were not significantly associated with depressive symptoms.
6.	Simpson, William, et al.^36^ Canada, 2016 Cohort	33 healthy pregnant women (18 to 45 years) in the third trimester of pregnancy (≥ 26 weeks gestation), free of major medical comorbidities (e.g., diabetes, hypertension, or other inflammatory conditions), and nonsmokers. Depressive symptoms were assessed using EPDS. Mean age was 30.7 + 4 years	IL-6 and IL-10 emerged as significant predictors of postpartum EPDS scores. Regression coefficients were negative, indicating that a lower level of both cytokines during the third trimester of pregnancy was predictive of a higher postpartum EPDS score. No relationship was observed between postpartum EPDS score and TNF-α or CRP.
7.	Drozdowicz-Jastrzębska, Ewa, et al.^37^ Poland, 2023 Cross-sectional	A total of 119 women who gave birth at the First Faculty and Department of Obstetrics and Gynaecology of the Medical University of Warsaw in the period 2013–2016 were included in the study. The exclusion criteria were age under 18 years and unstable physical condition. Depressive symptoms were assessed using EPDS and HDRS. Mean age was 31 + 3.95 years	The difference between IL-6 and IL-10 levels in women with and without depression was not statistically significant.
8.	Miller, Emily, et al.^38^ United States, 2019 Cohort	35 postpartum women with depressive symptoms. Depressive symptoms were assessed using the HDRS. Mean age was 27.1 + 5.4 years	CRP levels were not significantly associated with HDRS score even after controlling for maternal BMI and total night sleep.
9.	Liu, Hao, et al.^39^ China, 2016 Cohort	296 pregnant women who were hospitalised within 48 hours after the delivery of a singleton, full-term (≥37 weeks of gestation) live-born infant. Exclusion criteria are women with intrauterine demise or with infants immediately admitted to the neonatal intensive care unit, and (4) women with depression during or pre-pregnancy depression (according to their medical records or self-report). Depressive symptoms were assessed using EPDS. Mean age was 30.5 + 2.2 years	The serum levels of Hs-CRP and IL-6 after delivery in women with depression were significantly higher than in women without depression.

**CES-D:** Center for Epidemiologic Study-Depression; **CXCL1:** C-X-C motif chemokine 1**; EPDS:** Edinburgh Postnatal Depression Scale; **FGF-23:** Fibroblast growth factor 23; **HDRS:** Hamilton Depression Rating Scale; **HGF:** Hepatocyte growth factor; **IL:** Interleukin; **KYN:** kynurenine; **SERO:** serotonin; **TNF:** Tumour Necrosis Factor; **TRANCE:** Tumor necrosis factor ligand superfamily member; **TRY:** Tryptophan; **POMS**: Profile of Mood States; **PSS:** Perceived Stress Scale.

## Discussion

This systematic review found an inconsistent relationship between inflammatory cytokines and depressive symptoms in postpartum women. IL-1β was significantly elevated in postpartum women with depressive symptoms in two studies, suggesting its potential as a biomarker for detecting depression in postpartum women. However, findings on IL-6 were mixed; three studies reported an association, while others did not. IL-10 and TNF-α generally showed no significant association with PPD. These results suggest that while IL-1β may be relevant, more research is required to clarify the roles of other cytokines.

The results align with previous research indicating the involvement of inflammatory cytokines in depressive disorders, but underscore the variability of findings. Similar to earlier studies, IL-1β emerged as a more consistent marker, while IL-6 demonstrated conflicting results [24 - Osborne et al., 2019; 31 - Cheng & Pickler, 2014]. These inconsistencies are likely due to differences in study designs, sample sizes, and cytokine measurement techniques. Prior research has also highlighted mixed results for IL-6, reflecting the need for standardised methodologies to assess its role in postpartum depression [23 - Dunn et al., 2015].

The potential link between cytokines and PPD may involve several biological pathways. Hormonal fluctuations after childbirth, particularly the sudden decline in estrogen and progesterone, can trigger immune responses and increase cytokine levels. [12,29] Elevated levels of IL-1β and IL-6 may interfere with the serotonin and dopamine neurotransmitter systems, leading to mood disturbances. [13,30] Additionally, dysregulation of the hypothalamic-pituitary-adrenal (HPA) axis due to stress could enhance inflammation, exacerbating depressive symptoms. [14]

The included studies exhibited a range of strengths and weaknesses. A key strength was the use of validated depression scales like EPDS and CES-D, ensuring a reliable assessment of depressive symptoms. The potential for IL-1β to serve as a biomarker for postpartum depression could have significant clinical implications. If further validated, measuring IL-1β levels could help identify postpartum women at risk for developing depressive symptoms, facilitating early intervention. However, their clinical utility remains uncertain given the inconsistent findings on IL-6, IL-10, and TNF-α. Clinicians should continue to use comprehensive assessments of psychosocial factors, while cytokine measures could complement these in the future.

This review has several limitations. Many studies were limited by small sample sizes, which reduced the generalizability of their findings. The heterogeneity in cytokine measurement methods, as well as the timing of assessments, also contributed to inconsistent results. The risk of bias was moderate to high in some studies, further complicating the interpretation of the conclusions. Finally, the exclusion of non-English language studies may have omitted relevant research, reducing the scope of this review.

Future research should address the limitations identified in this review. Larger, more diverse sample sizes are necessary to improve the generalizability of findings. Additionally, studies should focus on longitudinal tracking of cytokine levels across the perinatal period to determine the timing of inflammatory responses in relation to depressive symptoms. Exploring the interaction between biological and psychosocial factors, such as stress, trauma, and social support, will provide a more comprehensive understanding of the appearance of depressive symptoms in postpartum women.

## Conclusion

This systematic review highlights the potential role of IL-1β in postpartum depressive symptoms, while the evidence for IL-6, IL-10, and TNF-α remains inconclusive. Further high-quality studies are needed to clarify the relationship between inflammatory cytokines and postpartum depression and to explore their potential as biomarkers for clinical use.

### Key points

IL-1β is significantly higher in postpartum women with depressive symptoms.
IL-2 and TNF-α are not significantly different between those groups.
The relationship between IL-6 levels and depressive symptoms in postpartum women is still inconsistent.
The relationship between IL-10 levels and depressive symptoms in postpartum women favours no significant association.
The relationship between CRP levels and depressive symptoms in postpartum women favours no significant association.

## References

[1] Liu X, Wang S, Wang G (2022). Prevalence and risk factors of postpartum depression in women: A systematic review and meta-analysis. J Clin Nurs..

[2] Chen L, Ding L, Qi M, Jiang C, Mao XM, Cai WZ (2018). Incidence of and social-demographic and obstetric factors associated with postpartum depression: differences among ethnic Han and Kazak women of Northwestern China. PeerJ.

[3] Weldu A, Belachew A, Yilma M, Doherty T (2023). The relationship between postpartum depression and appropriate infant feeding practice in eastern zone of Tigray, Ethiopia: A comparative cross-sectional study. PLoS One..

[4] Hamdan A, Tamim H (2012). The relationship between postpartum depression and breastfeeding. Int J Psychiatry Med..

[5] Nugrahaeni MT, Untari NY, Veibiani NA (2022). Meta analysis: The effect of social support in preventing postpartum depression in postpartum mothers. J Epidemiol Public Health..

[6] Ahlqvist-Björkroth S, Axelin A, Korja R, Lehtonen L (2019). An educational intervention for NICU staff decreased maternal postpartum depression. Pediatr Res..

[7] Trumello C, Candelori C, Cofini M, Cimino S, Cerniglia L, Paciello M (2018). Mothers' depression, anxiety, and mental representations after preterm birth: A study during the infant's hospitalization in a neonatal intensive care unit. Front Public Health..

[8] Zheng J, Han R, Gao L (2024). Social support, parenting self-efficacy, and postpartum depression among Chinese parents: The actor-partner interdependence mediation model. J Midwifery Womens Health..

[9] Zlotnick C, Tzilos G, Miller I, Seifer R, Stout R (2016). Randomized controlled trial to prevent postpartum depression in mothers on public assistance. J Affect Disord..

[10] Upadhyay RP, Chowdhury R, Salehi Aslyeh, Sarkar K, Singh SK, Sinha B (2017). Postpartum depression in India: a systematic review and meta-analysis. Bull World Health Organ..

[11] Jankowska K, Woźniak PA (2020). Hormonal conditions of postpartum depression. Wiedza Medyczna..

[12] Melón LC, Hooper A, Yang X, Moss SJ, Maguire J (2018). Inability to suppress the stress-induced activation of the HPA axis during the peripartum period engenders deficits in postpartum behaviors in mice. Psychoneuroendocrinology.

[13] Payne JL, Maguire J (2019). Pathophysiological mechanisms implicated in postpartum depression. Front Neuroendocrinol..

[14] Hutchens BF, Kearney J (2020). Risk factors for postpartum depression: An umbrella review. J Midwifery Womens Health..

[15] Bondy E, Norton SA, Voss M, Marks RB, Boudreaux MJ, Treadway MT (2021). Inflammation is associated with future depressive symptoms among older adults. Brain Behav Immun Health..

[16] McFarland DC, Jutagir DR, Rosenfeld B, Pirl W, Miller AH, Breitbart W Depression and inflammation among epidermal growth factor receptor (EGFR) mutant nonsmall cell lung cancer patients. Psychooncology..

[17] Kiecolt-Glaser JK, Derry HM, Fagundes CP (2015). Inflammation: depression fans the flames and feasts on the heat. Am J Psychiatry..

[18] Slavich GM, Irwin MR (2014). From stress to inflammation and major depressive disorder: A social signal transduction theory of depression. Psychol Bull..

[19] Eisenberger NI, Berkman ET, Inagaki TK, Rameson LT, Mashal NM, Irwin MR (2010). Inflammation-induced anhedonia: Endotoxin reduces ventral striatum responses to reward. Biol Psychiatry..

[20] Felger JC, Li Z, Haroon E, Woolwine BJ, Jung MY, Hu X (2016). Inflammation is associated with decreased functional connectivity within corticostriatal reward circuitry in depression. Mol Psychiatry..

[21] Dooley LN, Kuhlman KR, Robles TF, Eisenberger NI, Craske MG, Bower JE (2018). The role of inflammation in core features of depression: Insights from paradigms using exogenously-induced inflammation. Neurosci Biobehav Rev..

[22] Haroon E, Daguanno AW, Woolwine BJ, Goldsmith DR, Baer W, Wommack EC (2018). Antidepressant treatment resistance is associated with increased inflammatory markers in patients with major depressive disorder. Psychoneuroendocrinology.

[23] Dunn AB, Paul S, Ware LZ, Corwin EJ (2015). Perineal injury during childbirth increases risk of postpartum depressive symptoms and inflammatory markers. J Midwifery Womens Health..

[24] Osborne LM, Yenokyan G, Fei K, Kraus T, Moran T, Monk C (2019). Innate immune activation and depressive and anxious symptoms across the peripartum: An exploratory study. Psychoneuroendocrinology.

[25] Guintivano J, Sullivan PF, Stuebe AM, Penders T, Thorp J, Rubinow DR (2018). Adverse life events, psychiatric history, and biological predictors of postpartum depression in an ethnically diverse sample of postpartum women. Psychol Med..

[26] Page MJ, McKenzie JE, Bossuyt PM, Boutron I, Hoffmann TC, Mulrow CD (2021). The PRISMA 2020 statement: An updated guideline for reporting systematic reviews. The BMJ.

[27] Seo HJ, Kim SY, Lee YJ, Park JE (2023). RoBANS 2: A revised risk of bias assessment tool for nonrandomized studies of interventions. Korean J Fam Med..

[28] Susskind B, McCormack C, Veron E, Gupta A, Brito N, Thomason M (2024). Exploring associations between postpartum depression, acute-phase inflammatory markers (CRP and AGP), and socioeconomic factors in mothers at 6 months postpartum. Biol Psychiatry..

[29] Stanescu A, Balalau D, Ples L, Paunica S, Balalau C (2018). Postpartum depression: Prevention and multimodal therapy. J Mind Med Sci..

[30] Osborne LM, Gispen F, Sanyal A, Yenokyan G, Meilman S, Payne JL (2017). Lower allopregnanolone during pregnancy predicts postpartum depression: An exploratory study. Psychoneuroendocrinology.

[31] Cheng CY, Pickler RH (2014). Perinatal stress, fatigue, depressive symptoms, and immune modulation in late pregnancy and one month postpartum. Sci World J..

[32] Bränn E, Fransson E, White RA, Papadopoulos FC, Edvinsson Å, Kamali-Moghaddam M (2020). Inflammatory markers in women with postpartum depressive symptoms. J Neurosci Res..

[33] Groer ME, Jevitt C, Ji M (2015). Immune changes and dysphoric moods across the postpartum. Am J Reprod Immunol..

[34] Corwin EJ, Johnston N, Pugh L (2008). Symptoms of postpartum depression associated with elevated levels of interleukin-1 beta during the first month postpartum. Biol Res Nurs..

[35] Sha Q, Madaj Z, Keaton S, Escobar Galvis ML, Smart L, Krzyzanowski S (2022). Cytokines and tryptophan metabolites can predict depressive symptoms in pregnancy. Transl Psychiatry..

[36] Simpson W, Steiner M, Coote M, Frey BN (2016). Relationship between inflammatory biomarkers and depressive symptoms during late pregnancy and the early postpartum period: a longitudinal study. Revista Brasileira de Psiquiatria..

[37] Drozdowicz-Jastrzębska E, Mach A, Skalski M, Januszko P, Jabiry-Zieniewicz Z, Siwek M (2023). Depression, anxiety, insomnia and interleukins in the early postpartum period. Front Psychiatry..

[38] Miller ES, Hoxha D, Pinheiro E, Grobman WA, Wisner KL (2019). The association of serum C-reactive protein with the occurrence and course of postpartum depression. Arch Womens Ment Health..

[39] Liu H, Zhang Y, Gao Y, Zhang Z (2016). Elevated levels of Hs-CRP and IL-6 after delivery are associated with depression during the 6 months post partum. Psychiatry Res..

